# Synthesis and Evaluation of Aquatic Antimicrobial Peptides Derived from Marine Metagenomes Using a High-Throughput Screening Approach

**DOI:** 10.3390/md23040178

**Published:** 2025-04-20

**Authors:** Kaiyue Wu, Guangxin Xu, Yin Tian, Guizhen Li, Zhiwei Yi, Xixiang Tang

**Affiliations:** 1 College of Marine Sciences, Fujian Agriculture and Forestry University, Fuzhou 350002, China; wky991130@163.com; 2 Key Laboratory of Marine Genetic Resources, Third Institute of Oceanography, Ministry of Natural Resources, Xiamen 361005, China; xuguangxin@tio.org.cn (G.X.); tianyin@tio.org.cn (Y.T.); liguizhen.ok@163.com (G.L.)

**Keywords:** machine learning, cell-free protein expression, marine antimicrobial peptides, macro-genomes

## Abstract

Bacterial diseases cause high mortality and considerable losses in aquaculture. The rapid expansion of intensive aquaculture has further increased the risk of large-scale outbreaks. However, the emergence of drug-resistant bacteria, food safety concerns, and environmental regulations have severely limited the availability of antimicrobial. Compared to traditional antibiotics, antimicrobial peptides (AMPs) offer broad spectrum activity, physicochemical stability, and lower resistance development. However, their low natural yield and high extraction costs along with the time-consuming and expensive nature of traditional drug discovery, pose a challenge. In this study, we applied a machine-learning macro-model to predict AMPs from three macrogenomes in the water column of South American white shrimp aquaculture ponds. The AMP content per megabase in the traditional earthen pond (TC1) was 1.8 times higher than in the biofloc pond (ZA1) and 63% higher than in the elevated pond (ZP11). A total of 1033 potential AMPs were predicted, including 6 anionic linear peptides, 616 cationic linear peptides, and 411 cationic cysteine-containing peptides. After screening based on structural, and physio-chemical properties, we selected 10 candidate peptides. Using a rapid high-throughput cell-free protein expression system, we identified nine peptides with antimicrobial activity against aquatic pathogens. Three were further validated through chemical synthesis. The three antimicrobial peptides (K-5, K-58, K-61) showed some inhibitory effects on all four pathogenic bacteria. The MIC of K-5 against Vibrio alginolyticus was 25 μM, the cell viability of the three peptides was higher than 70% at low concentrations (≤12.5 μM), and the hemolysis rate of K-5 and K-58 was lower than 5% at 200 μM. This study highlights the benefits of machine learning in AMP discovery, demonstrates the potential of cell-free protein synthesis systems for peptide screening, and provides an efficient method for high-throughput AMP identification for aquatic applications.

## 1. Introduction

Bacterial diseases considerably hinder the sustainable and healthy growth of aquaculture, with antibiotics commonly used for disease prevention and control [1]. Environmental pressures and intensive aquaculture practices increase the susceptibility of aquatic animals to pathogens. However, the misuse of antibiotics has escalated the prevalence of antibiotic-resistant genes (ARGs) and antibiotic-resistant bacteria (ARB) in the environment. Research shows that approximately 20% of Vibrio strains isolated from inland saline aquaculture sites exhibit resistance to multiple antibiotic classes [2]. The development of bacterial resistance has far outpaced the discovery of new antibiotics, posing a major threat to human health and edging us toward a post-antibiotic era [3]. Compared to conventional antibiotics, AMPs—low-molecular-weight peptides—offer superior antibacterial activity, a broader spectrum, and a lower likelihood of inducing resistance mutations in target strains [4]. While most of the developed and used AMPs originate from long-term research and the development of terrestrial microorganisms, the microbial resources contained in the oceans, which account for more than 70% of the Earth’s surface area, have not yet been fully exploited. In the past 20 years, ocean prospecting technology, high-throughput sequencing technology, and macro-genomics research have been advancing rapidly, and many genetic resources with potential applications have been discovered [5]. In this paper, South American white shrimp aquaculture water was selected for the prediction of AMPs due to the presence of a rich and diverse microbial community in the aquaculture ponds, as well as the frequent use of antibiotics in modern aquaculture. The study of AMPs in aquaculture pond water samples has the potential to enhance the efficiency and economic viability of aquaculture, whilst concurrently reducing antibiotic usage and promoting the sustainable development of aquaculture.

Antimicrobial peptides found from nature are costly to produce, have low bioactivity, and may produce hemolytic and cytotoxic by-products, all of which have severely hampered their development [6]. In the contemporary era, numerous methodologies have been developed to artificially modify AMPs. These methodologies cover a variety of techniques, including altering the number of disulfide bonds, truncating peptide sequences, making amino acid substitutions, preparing heterodimeric peptides, designing computer molecular simulations, and creating minimized AMPs from scratch to enhance their accessibility [7].

The advent of bioinformatics and artificial intelligence-driven macromodeling has yielded new concepts in antimicrobial peptide design. Machine learning, a powerful tool for information processing, has attracted considerable attention in the realm of antimicrobial peptide design and development [8]. Some machine-learning models use deep learning (DL), a process that involves the implementation of complex transformations via deep neural networks. These transformations are designed to extract latent features and generate predictions from complex input and model data, including images and biomolecules. Machine learning, especially deep learning, has been widely used in various fields of computational biology, such as genome research [9,10], biomolecular structure modeling [11,12], drug discovery, drug development [13,14], and medical data analysis [15].

The application of machine learning to AMPs has greatly accelerated the discovery and optimization of new AMPs. For example, AMPs mined from marine biofilm bacteria by combining Ribo-seq and deep learning models showed significant inhibition of drug-resistant strains [16]. Machine learning has also revealed unprecedented bioactive relationships through in-depth data analysis and pattern recognition, providing innovative and efficient solutions for future challenges of antibiotic resistance [17]. Despite its enormous potential in AMP discovery, the broad application of this approach is limited by the lack of convenient methods to produce and screen more AMP candidates in medium-to-high throughput.

A potential method for enhancing AMP expression involves transitioning from chemical synthesis to DNA-based bioproduction approaches. However, the heterologous expression of AMPs in microorganisms comes with several challenges. These include the need for cloning, production, and purification—all of which are time-consuming and labor-intensive processes—as well as the possible risk of lethality to the production strain during induction [18]. The *E. coli* lysate-based cell-free protein synthesis (CFPS) system, first documented in 1961, has been identified as a potential solution to these challenges [19]. This system provides transcription–translation functions for synthesizing specific proteins for antigen detection [20]. Additionally, CFPS systems can be used to prepare gene line-associated sensors for detecting nucleic acid molecules and ions, etc. [21]. Importantly, CFPS offers the potential for high-throughput, cost-effective production, and the detection of bioactive peptides. In a noteworthy study, Amir Pand’s team used deep learning to engineer novel AMPs from the ground up. They successfully screened six new AMPs showing broad-spectrum activity against multidrug-resistant pathogens using CFPS [22].

In this study, we used a machine-learning macro-model to predict three macro-genomes obtained from the pond water of South American white shrimp farms. We further screened for peptides that possessed inhibitory activity against aquatic pathogens, low toxicity, and low hemolytic activity, selecting ten candidate antimicrobial peptide sequences with diverse morphological structures. By utilizing synthetic biology techniques, we created a rapid high-throughput screening channel for AMPs, based on a cell-free protein expression system. Of these peptides, all showed antimicrobial activities except for K-35. We further validated the antimicrobial activity of three peptides through chemical synthesis. These results underscore the potential of integrating machine learning with cell-free protein expression systems for mining AMPs, thus validating a viable approach for screening marine drug candidates.

## 2. Results

### 2.1. Prediction and Screening of AMPs in the Macrogenome

It has been shown that closed shrimp ponds serve as breeding grounds for the proliferation of ARB and ARG in aquatic environments [23]. This paper selected three mature and stable farms in Zhangzhou City, Fujian Province from which to collect samples of farmed seawater. The three shrimp farms, all in the late stage of South American white shrimp aquaculture, employed different cultivation methods: earthen pond aquaculture, elevated-tank aquaculture with a membrane, and biofloc factory aquaculture. The samples TC1, ZA1, and ZP11 were from earthen pond culture, biofloc factory culture, and high-level tank culture, respectively. The macro-genomes from these samples were predicted using Macrel.

The macrogenome TC1 had a total of 443,625 contigs with a total length of 976.00 Mbp, including 296,903 small open reading frames (smORFs)and 602 AMPs. Macrel calculated the AMP density in the sample to be 0.617 AMPs/Mbp. The macrogenome ZA1 contained 173,048 contigs with a total length of 592.29 Mbp, 95,403 smORF, and 202 AMPs. Subsequently, the density of AMPs in the sample was calculated to be 0.341 AMP/Mbp.ZP11 and they had a total of 188,322 contigs with a total length of 540.99 Mbp, 113,585 smORFs, and 253 AMPs. Macrel calculated the AMP density in the sample to be 0.468 AMPs/Mbp.

**Figure 1:**
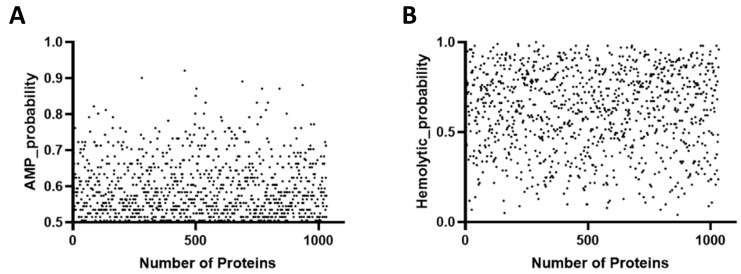
Likelihood coefficients and hemolysis coefficients for antimicrobial peptides. (**A**) The likelihood coefficients for antimicrobial peptides in the macrogenome were predicted by Macrel. A total of 1033 peptides with scores greater than 0.5 were predicted to be antimicrobial peptides. (**B**) Hemolysis coefficients for 1033 candidate antimicrobial peptides. Coefficients greater than 0.5 were considered to be at possible risk of hemolysis.

The AMP content per million bases was 1.8 times higher in the traditional earthen pond (TC1) than in the biofloc pond (ZA1) and 63% higher than in the elevated pond (ZP11). This discrepancy could be linked to the ponds’ geographical location, culture method, and microbial competition. Duplicate sequences were removed, and those with a score of 0.5 or higher were considered likely AMPs (Figure 1A). This process resulted in the prediction of 1033 candidate AMPs, including six anionic linear peptides, 616 cationic linear peptides, and 411 cationic cysteine-containing peptides. The hemolysis coefficients of the 1033 candidate peptides were scattered between 0 and 1, with values greater than 0.5 considered as potential risks for hemolysis (Figure 1B).

**Figure 2:**
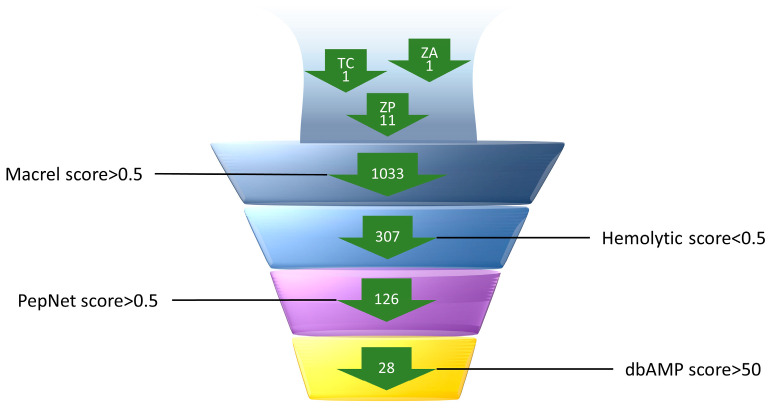
This figure is intended to provide a representation of the virtual screening results of AMPs for the present experiment to demonstrate the virtual screening process. The simulation was executed in a stepwise manner, commencing from the top and concluding at the base, following the stipulated screening conditions. The values indicated within the figure signify the number of peptides that were screened at each respective step.

The 1033 AMPs forecasted by Macrel were further screened by eliminating those predicted to be hemolytic, leaving 307 AMPs. For a more accurate screening of peptides with high antimicrobial activity and to reduce the number of false positives, the peptides were further sifted using PepNet and dbAMP2.0. A total of 126 candidate peptides with scores greater than 0.5, which were considered to be antimicrobial, were screened using PepNet. Subsequently, further prediction with dbAMP screened 28 candidate antimicrobial peptides with an anti-Gram-positive bacteria score of greater than 50 and an appropriate molecular size. The specific screening process is shown in Figure 2.

### 2.2. Physicochemical Properties of Candidate AMPs

The physicochemical properties of AMPs have been shown to relate to their antimicrobial actions. Such properties include amino acid composition, peptide length, the presence of positively charged residues, lipid composition, hydrophobic characteristics, and the molecule’s net charge. To analyze the method’s reliability in a representative way and screen valuable peptides using optimal parameters, we predicted the physicochemical properties of AMPs by combining APD3 and ProtParam. We later selected a total of ten post-selected AMPs with different structures to proceed with further experiments (Table 1 and Table 2).

The high net positive charge and amphiphilicity of AMPs suggest that they are capable of targeting negatively charged bacterial cell walls. The high affinity of AMPs for cell membranes likely results from this mechanism, contributing to the overall amphiphilicity of most AMPs. This infers that membrane interactions extend beyond α-helical peptides [24]. In our experiments, the candidate AMPs had charges between +3 and +5, hydrophobic cation ratios between 33% and 42%, and most isoelectric points between 9 and 12, and like the known AMPs, they had suitable charges with hydrophobicity and good water solubility. The aliphatic index of a protein, defined as the relative volume occupied by aliphatic side chains (that is, alanine, valine, isoleucine, and leucine), has been shown as a positive lement in the thermostability increase of a globular protein. The grand average of hydropathicity (GRAVY) was determined by comparing the sum of the hydrophilic values of all the amino acids in a sequence to the amino acid count. A negative value signifies superior hydrophilicity, while a positive value suggests enhanced hydrophobicity. The potential protein interaction index proposed by Boman (2003) was calculated based on the protein’s amino acid sequence. This index, equal to the sum of the solubility values for all residues in a sequence, might provide a general estimate of the peptide’s potential to bind to membranes or other protein receptor. To normalize it, the index is divided by the number of residues [25]. The hydrophobic cation ratio, also known as the amphiphilic equilibrium, greatly impacts antimicrobial activity and selectivity. The amphiphilic conformation encourages the separation of a cation’s hydrophobic and hydrophilic side chain. In light of the bacterial membrane’s structure, the amphiphilic conformation is integral to the physical interaction with the membrane. This makes it challenging to develop resistance [26].

### 2.3. The Rapid Synthesis and Screening of AMPs Is Based on Cell-Free Expression

A novel experimental pipeline for high-throughput synthesis and the testing of AMPs in a 100-well format was devised. This pipeline builds upon a prior AMP screening pipeline developed with a cell-free expression system [22]. This system uses a linear DNA template that integrates a T7 promoter and a RBS to start transcription (TX) and translation (TL), followed by the AMP coding region and a T7 terminator. Adding DNA templates (final concentration 300 ng) directly to 10 µL of the cell-free TX-TL system enables amp production within 16 h. To evaluate the antimicrobial activity of the peptides produced in vitro, 10 µL of the cell-free mixture was incorporated into a final volume of 50 µL of bacterial cultures. OD600 measurements were recorded after 24 h, and peptides that inhibit growth, indicating antimicrobial activity, could be identified. This system applies linear DNA and eliminates the need for laborious cloning or peptide purification steps. Additionally, the cell-free reaction temperature can be raised to accelerate the process if the experiment requires it.

**Figure 3:**
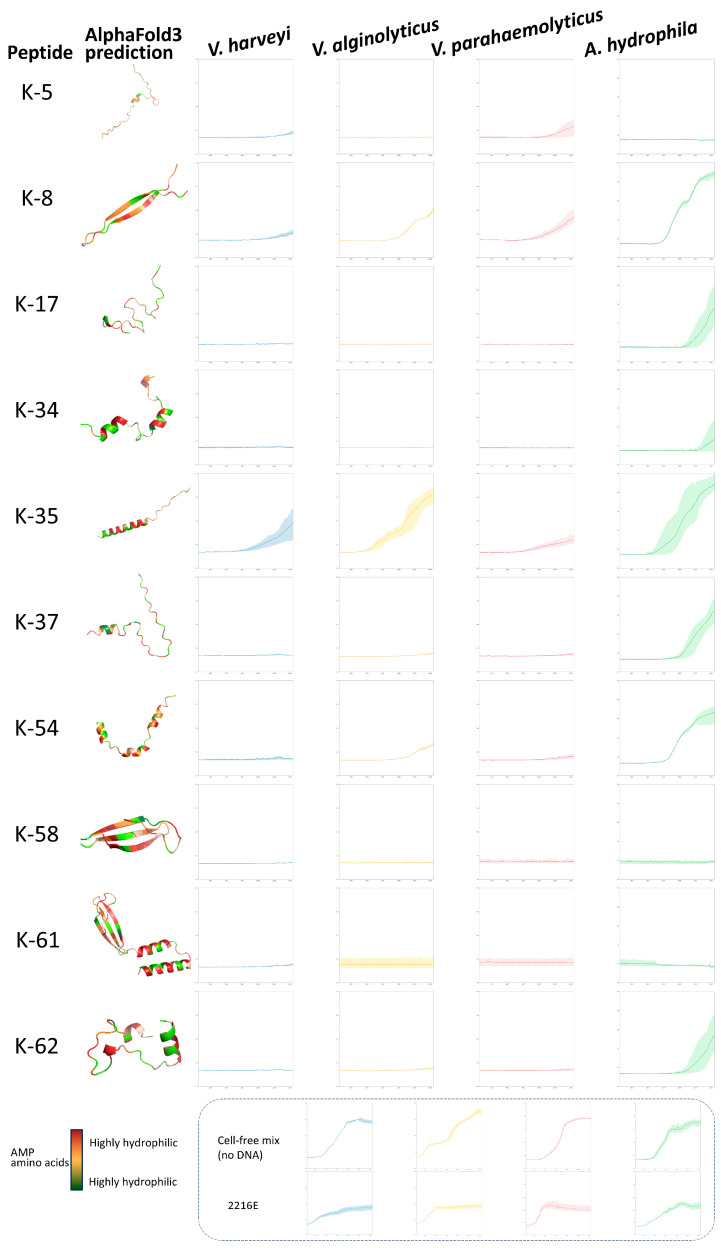
The structures of functional AMPs predicted by AlphaFold3 and the associated growth slowing/stopping curves for *V. harveyi*, *V. alginolyticus*, *V. parahaemolyticus*, and *A. hydrophila* are presented. All AMPs were produced using CFPS and were not peptide-purified before activity testing. OD600 over time 4–24 h growth curves (*n* = 3 independent experiments) with error bars as standard deviation.

The OD600 was measured at 10 min intervals. It was found that the group without the cell-free expression system or a DNA template had a higher OD600. In all tests, the AMPs K-35 did not significantly inhibit any of the other three bacteria, except for *Vibrio parahaemolyticus* in the early phase. In the inhibition test against *Vibrio harveyi*, K-17, K-34, K-37, K-54, K-58, and K-61 demonstrated effective inhibition within 24 h. The OD600 began to rise after 20 h in the groups of K-5, K-8, and K-62. In the inhibition test against *Vibrio alginolyticus*, K-5, K-17, K-58, and K-61 were the most effective within 24 h, and the rest of the AMPs inhibited the growth of *Vibrio alginolyticus* in the preliminary stage. In the inhibition test against *Vibrio parahaemolyticus*, all of the AMPs utilized demonstrated the capability to impede its growth in the initial stages. Several of the AMP test groups exhibited an increase in OD600 in the middle and late stages. Except for K-5, K-58, and K-61, which consistently exhibited the effective inhibition of *Aeromonas hydrophila* within 24 h, the OD600 of the remaining antimicrobial peptide test groups increased over time (Figure 3). We also chose two time points, 12 h and 24 h, to analyze for statistical significance using ANOVA (Figure S1).

### 2.4. Inhibition of V. harveyi, V. alginolyticus, V. parahaemolyticus, and A. hydrophila by Three AMPs

**Figure 4:**
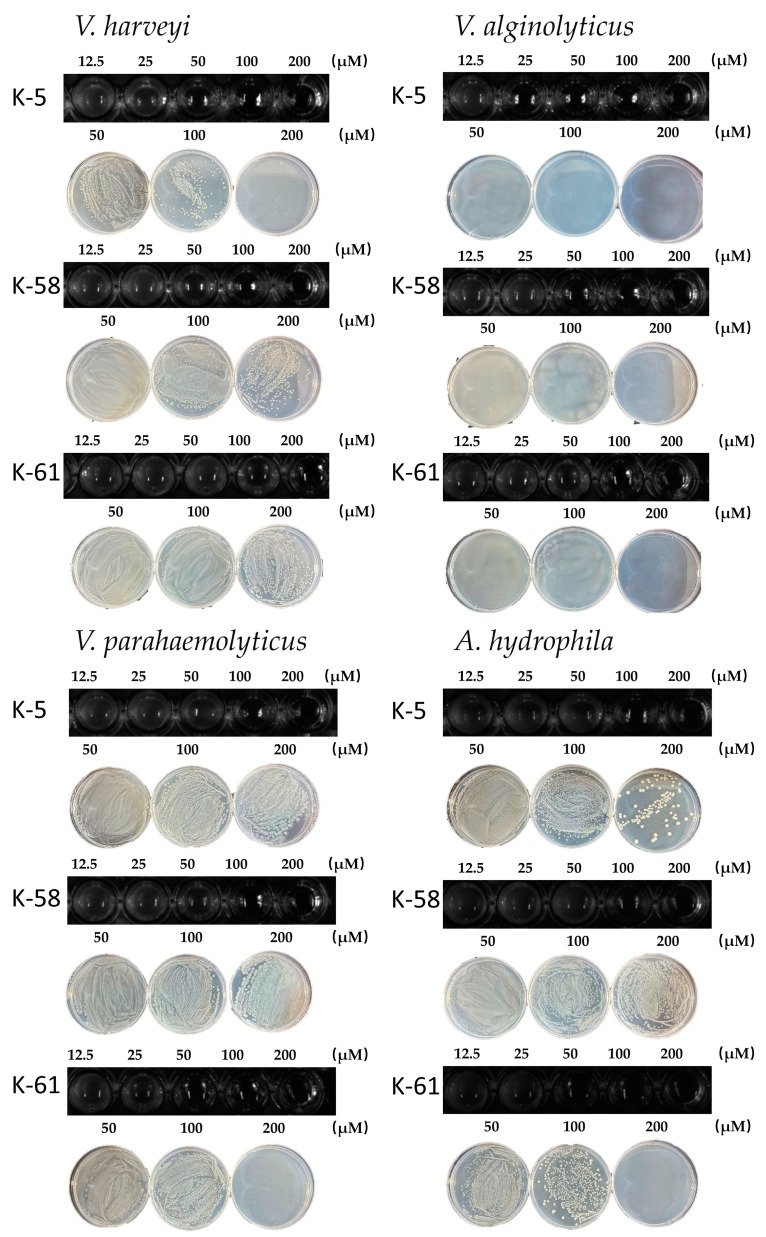
The MIC and MBC of three antimicrobial peptides against *Vibrio harveyi*, *Vibrio alginolyticus*, *Vibrio parahaemolyticus*, and *Aeromonas hydrophila* were measured in µM.

To evaluate the antibacterial activity of the three predicted peptides, we determined the MIC and MBC of the three peptides against *Vibrio harveyi*, *Vibrio alginolyticus*, *Vibrio parahaemolyticus* and *Aeromonas hydrophila* (Figure 4). As shown, all three peptides exhibited antimicrobial activity against aquatic pathogenic bacteria. Among them, K-5 showed significant inhibition against *V. alginolyticus* with the MIC of 25 µM and the MBC of 100 µM, *V. harveyi* with the MIC of 50 µM and the MBC of 200 µM, and weak inhibition against *V. parahaemolyticus* and *A. hydrophila*. K-58 inhibited Vibrio alginolyticus with the MBC of 200 µM. K-61 showed good inhibition of all three pathogenic strains except *Vibrio harveyi* with MICs in the range of 50–MP100 µM.

### 2.5. Evaluation of In Vitro Cytotoxicity and Hemolytic Activity

**Figure 5:**
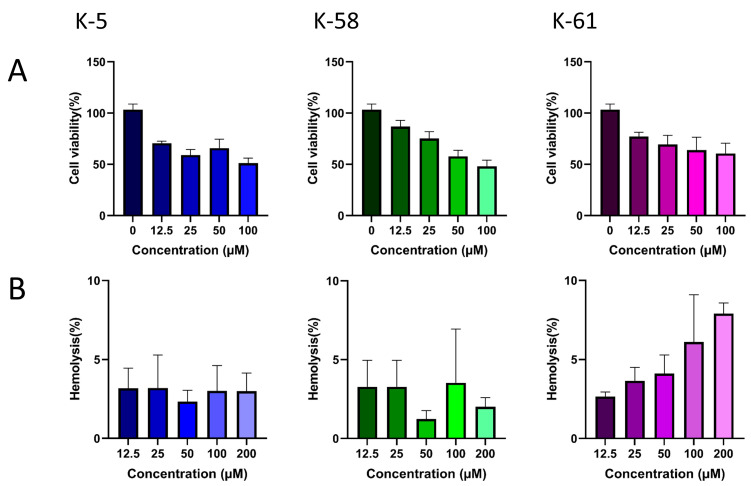
Cytotoxicity and hemolytic analysis of three peptides. (**A**) The cytotoxicity effects of antimicrobial peptides at different concentrations were tested on HEK293T cells. Peptide concentration (µM) was plotted on the *x*-axis and cell viability (%) on the *y*-axis (mean ± SD, *n* = 3). (**B**) Red blood cell hemolytic rates (%) were measured at varying concentrations of antimicrobial peptides (µM). Peptide concentration (µM) was plotted on the *x*-axis and hemolysis (%) on the *y*-axis (mean ± SD, *n* = 3).

We assessed the cytotoxicity of the three peptides using HEK293T cells (Figure 5A). Compared to the cell activity at a 0 µM concentration, HEK293T cell mortality rose progressively with the increasing peptide concentrations tested. Cell survival exceeded 70% at a concentration of 12.5 µM, but substantial cytotoxic effects were observed beyond this concentration. According to the criteria for determining the cytotoxicity level in ISO 10993-5:2009 (cell survival ≥ 70% is considered to be no risk of toxicity), the results of the present study indicate that the three antimicrobial peptides meet the criteria at low concentrations, and that further optimization is required to increase the safe concentrations. We evaluated the hemolytic activity of each peptide independently (Figure 5B). After co-incubation with erythrocyte suspension for 1 h, peptides K-5 and K-58 demonstrated less than 5% hemolysis, indicating minor hemolytic activity. In contrast, the hemolysis of peptide K-61 escalated with its increasing concentration. The three antimicrobial peptides we screened had a lower potential for haemolysis compared to some previously reported antimicrobial peptides with a haemolysis rate of <10% [27].

### 2.6. Effect of Peptide K-5 on V. alginolyticus and V. harvey Ultrastructure via Scanning Electron Microscopy

**Figure 6:**
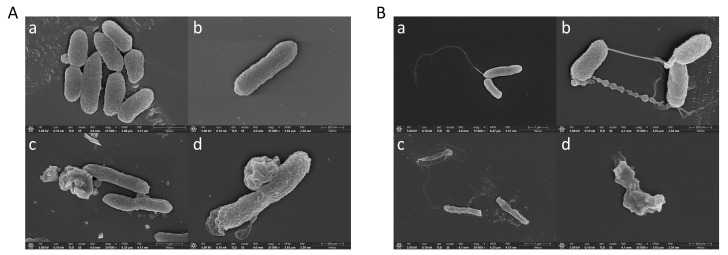
Effects of antimicrobial peptide K-5 on bacterial morphology and structure as observed by (**A**): Group of *V. alginolyticus*. (**a**,**b**) Treated with 0.01 M PBS (control); (**c**,**d**) Treated with 2× MBC K-5. (**B**): Group of *V. harveyi*. (**a**,**b**) Treated with 0.01 M PBS (control); (**c**,**d**) Treated with 2× MBC K-5.

The antimicrobial peptide K-5 was chosen for its ability to combat *V. alginolyticus* and *V. harveyi*. To determine the effect on cellular integrity, we utilized scanning electron microscopy to examine the morphology and structural integrity of the cell surface. As shown in Figure 6A, the electron microscopy results indicate that the control groups (Figure 6A(a,b)) demonstrated normal and undamaged *V. alginolyticus* cell morphology. However, the K-5 treated groups (Figure 6A(c,d)) showed signs of cell membrane damage, leakage of intracellular material, a rough, hazy, and concave cell surface, and the presence of holes or vesicles protruding from the cell surface. These observations imply a distinct alteration in the cells’ morphology and structure. The electron microscopy results (Figure 6B) illustrate that the control groups (Figure 6B(a,b)) showed normal and intact cell morphology of *V. harveyi*. On the other hand, the K-5-treated groups (Figure 6B(a,b)) revealed irregular changes in the morphology and structure of the bacterial cells, evidenced by visible damage, cell drying and crumpling, and the collapse of the cell structure.

## 3. Discussion

Whether in human healthcare, livestock production, or other areas, antibiotic resistance presents a serious challenge that we must be addressed immediately. Despite this looming threat, the development of novel antimicrobial agents continues to lag. However, in recent years, the rapid development of machine learning has led to its applications permeating various fields. Notably, the application of machine-learning techniques in AMP research has significantly expedited the development and production of AMPs, becoming a key driver in improving their yield and efficiency. This development has substantially advanced AMP research, particularly in the identification and generation of these peptides [28]. Machine-learning algorithms have proven instrumental in enabling the efficient screening and identification of potential antimicrobial peptide sequences. Similarly, generative models have been useful in assisting the design of new antimicrobial peptide molecules. These advances have not only enhanced the efficiency of antimicrobial peptide discovery but have also improved the accuracy of peptide design, thereby presenting new opportunities for their development and application [29]. Nine of the ten candidate peptides screened using the machine-learning model in this experiment showed an addition to good antimicrobial effects, illustrating the accuracy of machine learning.

Compared to other environments, the sea is vast and still contains a large amount of valuable, untapped information. Gradually, researchers are studying AMPs of marine origin, which exhibit novel sequences and intriguing structures. A prime example is the antimicrobial peptide URP20, derived from the Hong Kong oyster (*Crassostrea hongkongensis*), which has proven effective against various Gram-positive and Gram-negative food-borne pathogens, as well as Candida albicans, without displaying mammalian cell toxicity or mouse toxicity [30]. In the current study, a multi-step screening system was set up, incorporating a machine-learning prediction model to increase screening accuracy. The objective was to identify 28 candidate peptides from a plethora of gene fragments through a triple-screening method. Based on physicochemical property assessments, ten peptides were selected for subsequent experimentation. These peptides, ranging in length from 38 to 44 amino acids, were characterized by three primary features: an ample positive charge, excellent water solubility, and an effective membrane-penetrating structure. The candidate antimicrobial peptide K-5 is an irregularly coiled peptide, rich in glycine, which is the only amino acid without a side chain, and the high proportion of glycine gives the antimicrobial peptide a strong structural flexibility. Meanwhile, the simple structure of glycine reduces the protease recognition site, which may enhance the stability of the antimicrobial peptide [31]. The antimicrobial peptide K-58 has a classical β-fold structure, but chemically synthesized antimicrobial peptides lack the post-translational modifications of natural antimicrobial peptides (e.g., phosphorylation, acetylation) and it is difficult to synthesize complex spatial structures (e.g., disulphide bonding), which may contribute to the reduced activity of chemically synthesized K-58 [32]. The αβ structure of Psd1 achieves structural stability, target recognition flexibility, and efficient antimicrobial function through the clever combination of a rigid core and a dynamic region, which not only improves interaction with fungal membranes, but also confers broad-spectrum antimicrobial potential to the phytoalexin [33]. The K-61 predicted in this experiment also has both α-helical and β-folded structures, and this hybrid conformation may enhance its ability to penetrate cell membranes.

The absence of convenient methods for higher-scale production and screening of AMP candidates has rendered the chemical synthesis and heterologous expression of antimicrobial peptides in microorganisms time- and cost-intensive [34]. In this study, a fast, high-throughput screening approach based on a cell-free protein expression system capable of producing and assessing the impacts of these peptides on specific strains was implemented. This screening could be accomplished in 36 h or less. The cell-free protein expression system finds utility in various areas, including protein function research, swift diagnosis, optimizing metabolic pathways, and protein engineering [35]. However, there is still room for improvement in cell-free expression technology. In this experiment, the cell-free system was not optimized for targeting, the high number of heteroproteins in the system may have reduced the expression of target AMPs, and the antimicrobial peptide was too small (<10 kDa) to accurately quantify the amount of AMPs expressed in the cell-free system due to insufficient sensitivity. A series of ten candidate peptides exhibiting antibacterial activity were chosen for the present study. Among these, K-5, K-58, and K-61 displayed notable inhibitory effects against four different bacterial strains, without causing a significant increase in the OD600 value within a 24 h period. The method implemented is marked by its simplicity and efficiency, requiring minimal cloning steps and a shorter screening time. The chemically synthesized K-5, K-58, and K-61 were effective against four aquatic pathogens. The MIC of K-5 against *Vibrio alginolyticus* (VAL) was 25 µM, and the microbicidal concentration (MBC) was 100 µM. Irregularly coiled peptides are typically rich in arginine, tryptophan, or proline and usually lack a distinct secondary structure. However, they can interact with cell membranes through their linear arrangement of amino acids and can exert antimicrobial effects. Bovine neutrophil-derived indolicidin has demonstrated antibacterial, antiviral, antibiofilm, and wound-healing effects [36]. Electron microscopy revealed that the cell membranes of bacteria treated with K-5 ruptured, leading to the leakage of their contents.

In conclusion, the experiments conducted in this study demonstrate the effectiveness of machine learning in screening AMPs. The use of a cell-free system addresses the high cost and time-consuming nature of traditional methods. While further research is needed to assess the long-term safety and stability of the screened AMPs, the current findings suggest their potential to disrupt bacterial cells. This study offers a rapid, high-throughput strategy for developing novel antimicrobial pharmaceuticals.

## 4. Materials and Methods

### 4.1. Materials

*Vibrio alginolyticus* V181505 and *Vibrio parahaemolyticus* Vp519BL were kindly provided by the laboratory of Jimei University (Xiamen, China). *Vibrio harveyi* MCCC 1A20671 and *Aeromonas hydrophila* MCCC 1A00032 were obtained from the Marine Culture Collection of China (MCCC, Xiamen, China). The key components of the cell-free expression system transfer of *Escherichia coli* (tRNA) were purchased from Sigma Aldrich Ltd. (Shanghai, China). T7 RNA polymerase was procured from Sangong Bioengineering Co (Shanghai, China). All other chemicals and reagents were of analytical grade and were commercially purchased.

With regard to the macrogenomic sources, the soil pool sample TC1 was collected from Zhao’an County, Zhangzhou, with a longitude of E117.26 and dimension of N23.66; bioflocculation plant sample ZA1 was collected from Zhao’an County, Zhangzhou, with a longitude of E117.23 and dimension of N23.61; and high pool sample ZP11 was collected from Zhangpu County, Zhangzhou, with a longitude of E117.83 and dimension of N24.02. Each sample was taken in a volume of 2 L with a 0.22 filter membrane. A PE library was constructed using the NEBNext^®^ Ultra™ DNA Library Prep Kit, and after passing the quality control, bipartite sequencing was performed by the Illumina Hiseq X-ten platform. Low-quality sequences, junctions, and primers were removed using Trimmomatic (v0.36), and high-quality clean data were retained. contigs > 500 bp in length were screened by splicing based on the principle of succinct de Bruijn graphs using MEGAHIT (v1.0.6).

### 4.2. Prediction and Screening of Candidate AMPs

Macrel is an end-to-end process designed for the efficient screening of AMPs from genomic and macrogenomic data [37,38]. It addresses the high false-positive issue of traditional methods in short peptide prediction by combining 22 local and global features, such as free energy conversion, solvent accessibility, and charge. Using a random forest classifier to train the mode, Macrel simulates a low ratio of AMPs to non-AMPs (1:50) in real data, markedly improving prediction specificity (>99.8%) and accuracy. The process supports assembly, gene prediction, and the filtering of raw sequencing data, effectively reducing false positives and validating the recovery of high-quality AMP candidates in simulated and real macroeconomic data. Macrel provides opensource tools and online servers for the discovery of novel AMPs in clinical and ecological studies. Additionally, dbAMP 2.0 [39] stands out for its rich database resources and structural prediction capabilities, while PepNet [40] is notable for the high prediction accuracy and interpretability of its deep-learning models. The union of these three prediction models allows more precise screening for peptides with major antimicrobial activity, thereby decreasing false positives.

### 4.3. Prediction of Three-Dimensional Structures and Physicochemical Properties of Candidate AMPs

The three-dimensional (3D) structures of the candidate AMPs were predicted by AlphaFold3 [41] (https://alphafold3.org/, accessed on 21 October 2024). Visual analysis and renders used PyMOL [42] (Version 2.5.0, Schrödinger Inc., New York, NY, USA). The physicochemical properties of candidate AMPs were predicted using ProtParam [43] (https://web.expasy.org/protparam/, accessed on 22 September 2024) and APD3 [44] (https://aps.unmc.edu/prediction, accessed on 22 September 2024).

### 4.4. Construction of a Cell-Free Expression System

*Bacterial culture: Escherichia coli* BL21 (DE3) was streaked onto a 2× YT agar plate and incubated at 37 °C overnight. A single colony was then inoculated into 50 mL of 2× YT medium and cultured at 37 °C with agitation at 200 rpm for 12–16 h.

The following day, a 3× dilution of the overnight culture was prepared to measure the optical density at 600 nm (OD600). For this, 300 µL of bacterial suspension was mixed with 2.7 mL of fresh medium. The culture was then inoculated into 500 mL of 2× YT medium per bottle to achieve an initial OD600 of 0.1, and was incubated at 37 °C with agitation at 220 rpm. OD600 was monitored every 30 min after the first hour. When OD600 reached 0.6–0.8, isopropyl β-D-1-thiogalactopyranoside (IPTG) was added to a final concentration of 1 mM to induce protein expression. Cultivation continued until OD600 reached 1.8–2.7, at which point cells were harvested by centrifugation at 5000× *g* for 10 min at 4 °C. The bacterial pellet was collected, transferred to 50 mL centrifuge tubes, and maintained on ice. The pellets were washed three times by resuspension in 30 mL of S30 buffer, followed by centrifugation at 5000× *g* for 10 min at 4 °C. After the final wash, the pellet was transferred to 15 mL centrifuge tubes. The tubes were weighed before and after discarding the excess buffer. The samples were then snap-frozen in liquid nitrogen and stored at −80 °C.

*Cell lysis and protein extraction:* Frozen samples were thawed on ice for 30 min before resuspension in an equal volume of S30 buffer. The suspension was vortex-mixed thoroughly, and 1.4 mL of the mixture was transferred into 1.5 or 2 mL microcentrifuge tubes. Cell disruption was performed using an ultrasonic homogenizer set to 50% power (65 W, 20 kHz), applying sonication pulses of 15 s with 10 s intervals for a total sonication time of 45 s. Immediately after sonication, 4.5 µL of 1000 mM dithiothreitol (DTT) was added to achieve a final concentration of 2 mM, and the solution was mixed thoroughly. The lysate appeared light pink in color. The mixture was then centrifuged at 30,000× *g* for 10 min at 4 °C. The resulting supernatant was collected, snap-frozen in liquid nitrogen, and stored at −80 °C for further use.

*S30 buffer: 10 mM Tris, 14 mM magnesium glutamate; 60 mM potassium glutamate; 2 mM DTTSupplementary liquid preparation:* The supplementary solution was divided into three components, each prepared separately and then mixed proportionally. The concentration of each component is specified in Table 3.

The 20-amino-acid premix was prepared as follows: A solid KOH weighing 28.06 g was measured and poured into a beaker containing 60 mL of deionized water. It was then stirred with a glass rod until dissolved and allowed to cool to room temperature. The solution was transferred from the beaker into a 100 mL volumetric flask using the glass rod. The beaker was then rinsed with deionized water, and this rinse was added to the flask. The solution’s volume was finally adjusted to 100 mL using more deionized water, resulting in a 5 M KOH solution.

Each of the 20 amino acid powders were weighed after dissolving them in a 5 MKOH solution on ice, and then they were stored in the refrigerator at −20 °C. The final concentration of each amino acid reserve solution was 3 M.

A total of 50 μL of amino acid reserve solution was added to 5 mL deionized water in the following sequence: arginine, valine, tryptophan, phenylalanine, isoleucine, leucine, cysteine, methionine, alanine, asparagine, aspartic acid, glycine, glutamine, glutamic acid, histidine, lysine, proline, serine, threonine, tyrosine. Next, the pH was adjusted to 6.5 using glacial acetic acid. The final concentration of each amino acid was 15 mM, in a 20 amino acid premix. The solution was then filtered through a 0.22 μm filter membrane to remove bacteria, dispensed into 1.5 mL EP tubes, snap-frozen in liquid nitrogen, and stored at −80 °C for future use.

### 4.5. Rapid Synthesis and Screening of AMP Candidates

The linear gene template comprised T7 promoter (TAATACGACTCACTATAGG), the ribosome binding site (RBS) (TCTAGAGATTAAAGAGAGGAGAATAC), and T7 terminator (CTAGCATAACCCCTTGGGGGGCCTCTAAACGGGGTCTTGAGGGGTTTTTTG), and *E. coli* expression optimized AMP sequences. These linear DNA templates were supplied by GentleGen Biologicals (Suzhou, China). The cell-free AMP synthesis system, which was 10 µL in volume, contained 3.3 µL of fragmentation solution, 4.9 µL of supplementation solution, and 300 ng of linear DNA template, and ddH_2_O was added to make the total 10 µL. The reaction was undertaken at 30 °C and 180 rpm for 12 h for the synthesis of the candidate AMPs.

Referring to the previous literature, cell-free production and an activity test of AMPs method suitable for this experiment was designed [22]. On day one, *Vibrio alginolyticus*, *Vibrio parahaemolyticus*, *Vibrio harveyi*, and *Aeromonas hydrophila* were streaked and cultured on 2216E plates at 30 °C. The following day, individual colonies were separately picked for passaging in 2216E and were cultured at 30 °C with oscillation until OD600 ≈ 1.0. Cells were then diluted to 10^4^ CFU/mL in 2216E, and 40 µL of the diluted cells were added to the wells of a 100-well plate already containing 10 µL of a cell-free reaction mixture (which produced AMP). These cultures were mixed, and OD600 was monitored every 10 min on a microbial growth curve detector (FP-1100-c, Bioscreen C, Growth Curves, Telekatu 12 20360 Turku, Finland) at 30 °C for 24 h. Growth curves were then analyzed to determine whether the AMPs inhibited bacterial growth. Finally, we examined the plates by visually inspecting the microtitre plates after 24 h, and by visually analyzing the growth curves, looking for any cessation or slowing of growth over time compared to the control (OD600).

### 4.6. Chemical Synthesis of Antimicrobial Peptides K-5, K-58, and K-61

ChinaPeptides (QYAOBIO) Co., Ltd. (Shanghai, China) was contracted to synthetically produce the above-screened peptides through solid-phase synthesis. The peptides were then purified using high-performance liquid chromatography and verified by electrospray mass spectrometry to ensure a purity exceeding 95%.

### 4.7. Minimum Inhibitory Concentration and Minimum Bactericidal Concentration of Synthetic Peptides

The antimicrobial activity of synthetic peptides was determined using the conventional broth microdilution method [45]. *Vibrio harveyi*, *Vibrio alginolyticus*, *Vibrio parahaemolyticus*, and *Aeromonas hydrophila* were inoculated into a 2216E liquid medium and were incubated at 30 °C (180 rpm) for 16–24 h. The culture was diluted with a 2216E medium until the viable count reached ~10^6^ CFU/mL. The synthetic peptide was dissolved in phosphate-buffered saline (PBS) and was diluted in 96-well plates with a 2216E medium using a 2-fold gradient, with final concentrations of 200, 100, 50, 25, 12.5, 6.25, and 0 µM. An aliquot of 10 μL of bacterial culture was mixed with 90 μL of 2216E liquid medium and was incubated at 30 °C for 16–20 h. Sterile 2216E liquid medium served as a negative control. Following incubation, the minimum inhibitory concentration (MIC) of each antimicrobial peptide against bacteria was defined as the minimum reagent concentration preventing visible bacterial growth. Subsequently, an aliquot (100 μL) of bacterial culture was plated onto 2216E agar plates. After incubation for 24 h at 30 °C, the minimum bactericidal concentration (MBC) of each peptide against the bacteria was defined as the minimum concentration that inhibited bacterial cell growth on the plate.

### 4.8. Cytotoxicity Assay

HEK293T cells were cultured in DMEM (10% fetal bovine serum, 1% penicillin-streptomycin solution) at 37 °C within a 5% CO_2_ cell culture incubator, continuing until they reached the logarithmic growth phase. We assessed the cytotoxicity of the three peptides on the HEK293T cells using the Cell Proliferation and Cytotoxicity Assay Kit (CCK-8, BD0079, Bioworld, Nanjing, China). We inoculated the wells of a 96-well microtitre plate with 6 × 10^4^ cells and incubated it at 37 °C for 24 h, as described in previous articles [46]. We removed the supernatant from each well and added 100 μL of graded concentrations of antimicrobial peptide-containing DMEM (12.5, 25, 50, and 100 μM, *n* = 3 for each concentration). We used HEK293T cells cultured with DMEM (0 μM) as the control group. After incubating this for 12 h at 37 °C, we added CCK-8 solution (10 μL per well), then quantified cell metabolism after a further 2 h of incubation at 37 °C. We measured absorbance at 450 nm, calculating cell viability, which reflects the peptide’s cytotoxicity toward the cells, as follows:

Viability (%) = (OD450Treated − OD450Blank)/(OD450Untreated − OD450Blank) × 100%(1)

### 4.9. Hemolysis Assay

Blood from healthy rabbits was centrifuged at 1000 rpm for 10 min to isolate red blood cells. The supernatant was removed, and the erythrocytes were washed with PBS until the supernatant became clear. The resulting erythrocytes were then resuspended in PBS to prepare a 4% suspension for testing. Following a previously described method [47], the AMPs were diluted in PBS to final concentrations of 25, 50, 100, 200, and 400 μM using serial dilution. Subsequently, 100 μL of each peptide dilution was added to individual wells of a 96-well plate for the experimental group. The negative controls consisted of 100 μL of PBS, while the positive controls contained 10% Triton X-100, with three replicate wells for each concentration. The plates were incubated at 37 °C with 5% CO_2_ for 1 h. After incubation, they were centrifuged at 1500 rpm for 10 min, and the supernatant was transferred to a new 96-well plate. Optical density at 450 nm was measured using a spectrophotometer. Hemolysis was calculated using the following formula:

Hemolysis (%) = (OD450Experimental group − OD450Negative control)/(OD450Positive control − OD450Negative control)× 100%(2)

### 4.10. Effects of Antimicrobial Peptide K-5 on Bacterial Morphology and Structure as Observed by Scanning Electron Microscopy

Bacteria grown to the logarithmic phase in 2216E medium were harvested, centrifuged, and washed twice with sterile PBS after discarding the supernatant. The bacterial suspension was then treated with an antimicrobial peptide at a concentration of 2× MBC for 2.5 h, while the control group was treated with 0.01 M PBS for the same duration. Following treatment, the cells were centrifuged at 5000 rpm for 15 min, and the supernatant was removed. The pellet was then fixed with 2.5% glutaraldehyde at 4 °C for 4 h. A bacterial suspension was deposited dropwise onto a silicon wafer and allowed to stand for 15 min. The samples then underwent gradient dehydration using ethanol solutions: 30% for 5 min, 50% for 5 min, 70% for 10 min, 90% for 15 min, and 100% for 20 min. The 70% dehydration step was repeated twice, with all of the dehydration steps performed at 4 °C. The samples were then dried using a Leica EM CPD300 fully automated critical-point dryer. Finally, the gold coating was applied, and the specimens were imaged using a ThermoFisher Helios 5 UC focused ion beam scanning electron microscope.

## Figures and Tables

**Table 1:** Sequence information, structure prediction, and molecular weight of ten candidate antimicrobial peptides screened by machine learning.

Peptide	Sequence	Structure	Molecular Weight
K-5	QPYGSQGFYGQRKWGNGQGVPLSQSNGLGGRGGGGGQRLVSKCL	Rich in Gly	4494.980
K-8	NSYHIYRCTHCAVKQGGQPPSCNTLICPGKAS	Cys	3434.949
K-17	AECADLRGRRGGERRGILCGGEKGGSAGSGRVPILGRVG	Rich in Gly	3881.428
K-34	VLSLRRVALEDKGGLGPGAGFKKGLKVTAPARGQDKTA	helix	3863.511
K-35	VAVVVVGEVQRKKTGVALKQKQRAGSSGGGGGGRGGAEA	Rich in Gly	3707.203
K-37	FGIKGLKGEQLPEPKAPKGKYKSIGFGDLKESINDFFTNKK	helix	4556.257
K-54	ALVSDIIKNAKLDDSYGKNARGIPQTSDKLNGCSEKRAK	helix	4205.746
K-58	AKGKYCPYCKRPMFAQSEKQFPAGTEVIYTCTCGHKEKVFEDK	Cys	4948.750
K-61	LGGKKKKVLKAANDYVAKPRDEYEWRIYWRDMGKLLDDAR	helix	4797.546
K-62	LGLLKDLKARYPDAIIQGHRDFPNVKKSCPRFNAKEEYNF	helix	4693.403

**Table 2:** Physicochemical properties of 10 candidate peptides.

No.	Charge	pI	GRAVY	Aliphatic Index	Boman Index	Hydrophobic Ratio	Similar
K-5	+5	10.45	−0.816	48.64	1.56 kcal/mol	41.67%	41.67%
K-8	+3.5	8.89	−0.453	51.88	1.40 kcal/mol	37.14%	37.14%
K-17	+4	10.58	−0.467	72.56	2.41 kcal/mol	39.58%	39.58%
K-34	+5	10.55	−0.311	87.37	1.46 kcal/mol	40.48%	40.48%
K-35	+5	11.07	−0.284	74.87	1.38 kcal/mol	34.00%	34%
K-37	+4	9.63	−0.878	59.51	1.64 kcal/mol	37.78%	37.78%
K-54	+3	9.36	−0.848	77.69	2.60 kcal/mol	38.10%	38.1%
K-58	+3.25	8.78	−0.783	29.53	1.84 kcal/mol	36.36%	36.36%
K-61	+4	9.63	−1.115	73.25	2.82 kcal/mol	33.33%	33.33%
K-62	+3.25	9.36	−0.800	73.25	2.37 kcal/mol	36.36%	36.36%

**Table 3:** Components and the final concentration of make-up solution.

Salt		1 × Buffer		Nucleotide Solutions and Other	
HEPES	50 mM	tRNA	0.2 mg/mL	GTP	3 mM
Potassium glutamate	90 mM	CoA	0.26 mM	ATP	1 mM
Magnesium glutamate	15 mM	cAMP	0.75 mM	UTP	1 mM
		folinic acid	0.068 mM	CTP	1 mM
		NAD	0.33 mM	PEG8000	2%
		spermidine	0.66 mM	T7 RNA polymerase	200 U
		3-PGA	30 mM	20AA	1.5 mM each

## Data Availability

The original contributions presented in the study are included in the article; further inquiries can be directed to the corresponding authors.

## Supplementary Materials

The following supporting information can be downloaded at: https://www.mdpi.com/article/10.3390/md23040178/s1. Figure S1: Comparison of OD600 at 12 h, using one-way ANOVA.
