# Supplementary material for: Synthesis and Evaluation of Aquatic Antimicrobial Peptides Derived from Marine Metagenomes Using a High-Throughput Screening Approach
Source: Mar Drugs. 2025 Apr 20;23(4):178. doi: 10.3390/md23040178 (PMC12028987; doi:10.3390/md23040178)
Supplement: Supplementary file 1 [file marinedrugs-23-00178-s001.zip › marinedrugs-3567642-supplementary.pdf]

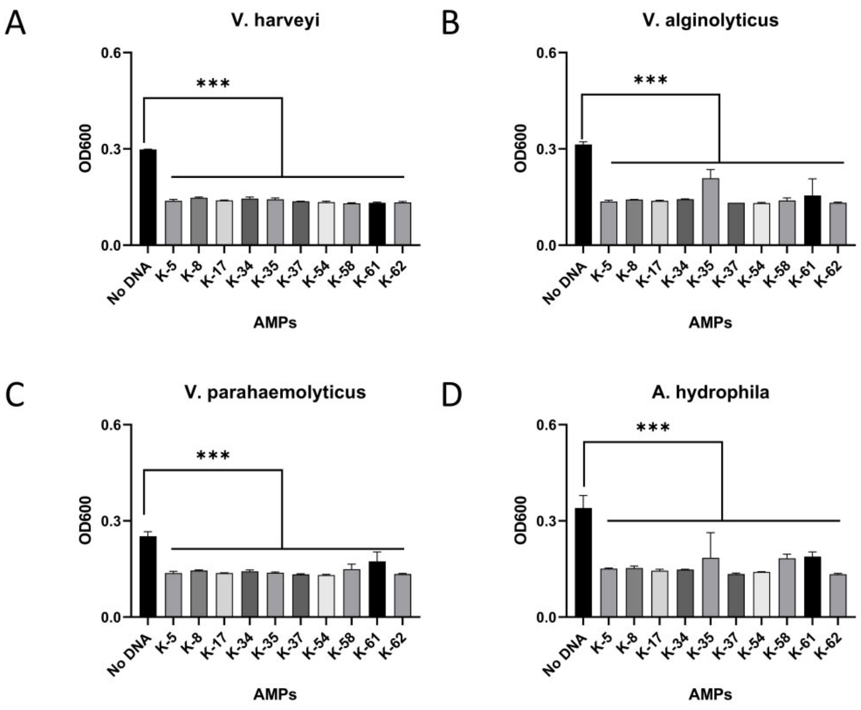

Figure S1. Comparison of OD600 at 12h, using one-way ANOVA. (A)Comparison of OD600 of different groups of *V. harveyi* at 12h.(B)Comparison of OD600 of different groups of *V. alginolyticus* at 12h. (C)Comparison of OD600 of different groups of *V. parahaemolyticus* at 12h. (D)Comparison of OD600 of different groups of *A. hydrophila* at 12h.
